# Effects of “trigeminal training” on trigeminal sensitivity and self-rated nasal patency

**DOI:** 10.1007/s00405-018-4993-5

**Published:** 2018-05-09

**Authors:** Anna Oleszkiewicz, Timo Schultheiss, Valentin A. Schriever, Jana Linke, Mandy Cuevas, Antje Hähner, Thomas Hummel

**Affiliations:** 1 Smell and Taste Center, Department of Otorhinolaryngology, TU Dresden, Fetscherstrasse 74, 01307 Dresden, Germany; 2 Institute of Psychology, University of Wroclaw, Wroclaw, Poland

**Keywords:** Trigeminal nerve, Trigeminal training, Trigeminal sensitivity, Nasal patency

## Abstract

**Purpose:**

Patients with the feeling of a congested nose not always suffer from an anatomical obstruction but might just have a low trigeminal sensibility, which prevents them from perceiving the nasal airstream. We examined whether intermittent trigeminal stimulation increases sensitivity of the nasal trigeminal nerve and whether this effect is accompanied by subjective improvement of nasal breathing.

**Method:**

Thirty-five patients (M_age_ = 58.4 years; SD = 14.8; Min_age_ = 21 years; Max_age_ = 79 years; 43% females) and 30 healthy controls (M_age_ = 36.7 years, SD = 14.5; Min_age_ = 20 years; Max_age_ = 73 years; 60% females) participated in a study comprised of two sessions separated by “trigeminal training”. During each session, trigeminal sensitivity towards CO_2_, trigeminal lateralization abilities and ratings of nasal patency were assessed. Age and training compliance were controlled.

**Results:**

“Trigeminal training” had a positive effect on trigeminal sensitivity in both groups, (*p* = .027) and this effect depended on the training compliance (*p* < .001). “Trigeminal training” had no effect on lateralization abilities of the subjects (*p* > .05). Ratings of nasal patency increased in patients (*p* = .03), but not in controls.

**Conclusions:**

“Trigeminal training” consisting of intermittent presentation of diverse stimulants leads to an increase of trigeminal sensitivity, but this effect depended on the training compliance. Importantly, in patients, this training is also associated with an increase in self-rated nasal patency.

## Introduction

The trigeminal nerve is involved in various chemosensory processes. Among many other functions, in the nasal cavity, it signals sensations of burning or stinging [1, 2], enables to lateralize odorants [3–5] and registers the nasal airflow [6–9]. The trigeminal nerve regulates a wide range of reactions, such as changes in respiratory rate/depth, changes in nasal secretion, changes in nasal airflow resistance, or sneezing. The nasal trigeminal sensitivity to irritants is higher in the anterior part as compared to the posterior part of the nose [10] and holds a protective function of the whole respiratory system [11–14]. Furthermore, involvement of trigeminal receptors during smelling increases chances that the source of stimuli will be correctly localized [4, 15–20]. Overall, the trigeminal nerve not only plays an important role in the subjectively felt nasal patency but also influences human abilities to navigate in the environment.

Patients complaining of lowered trigeminal sensitivity (often reported as a feeling of congested nose) are typically advised to have a surgical intervention. However, there is empirical evidence suggesting that trigeminal sensitivity does not improve with surgical interventions such as sinus surgery [21]. In fact, a subjectively congested nose may not exclusively have an anatomical origin but might also result from insensitivity of the nasal trigeminal nerve. In this context, it seems possible that the feeling of a decreased patency of the nasal cavity may stem from decreased trigeminal sensitivity [22, 23].

The subjective nasal patency was well-researched using menthol lozenges and menthol vapor, that once applied, are activating receptors in the respiratory epithelium. As a consequence, people report a better nasal patency and an impression of coolness, although no objective widening of the nasal cavity is observed or any significant temperature differences [7]. Other studies confirmed that direct cooling of the nasal mucosa enables participants to experience subjectively highest patency as compared to inhaling cold air [9, 24]. It appears that the subjective feeling of a wide nose is neither related to the actual nasal resistance nor to the minimum airway cross-sectional area; however, it seems to correlate with mucosal cooling [9].

Today, patients complaining of low nasal patency are typically advised to have a surgical intervention. In light of the findings on nasal trigeminal sensitivity, this may not always be the best practice, as the essential feeling of a cool mucosa is mediated by the trigeminal nerve and seems not to be directly related to the nasal anatomy, and does not improve with surgical interventions such as sinus surgery [21]. Taken together, recent results suggest that the subjectively congested nose may not exclusively have an anatomical origin but might also result from insensitivity of the nasal trigeminal nerve. In this context, it seems possible that subjective patency of the nasal cavity may be regained by increasing the sensitivity of the nasal trigeminal nerves.

There is a scant evidence suggesting that trigeminal sensitivity changes after exposure to trigeminally active substances [25, 26]. However, these studies mostly concerned changes in sensitivity towards the specific substance that subjects were exposed to. In the study of Dalton et al. [25], participant’s exposure to trigeminal stimuli was prolonged and intense, thus the observed effect could reflect their desensitization rather than beneficiary effects of intermittent, balanced stimulation aimed to “train” trigeminal nerve. The aim of the current study was to investigate whether an intermittent stimulation with a range of trigeminally active odorants might increase trigeminal sensitivity. To this end, we employed the concept of “trigeminal training”, wherein participants exposed themselves to three different trigeminal stimuli four times daily. We expected to observe an increased performance of trigeminal function.

## Materials and methods

### Ethics statement

The study was performed in accordance to the Declaration of Helsinki on Biomedical Studies Involving Human Subjects. Informed written consent was obtained from all the participants. The study design and consent approach was approved by the University of Dresden Medical Faculty Ethics Review Board (EK45022016).

### Participants

We determined the sample size by utilizing G*Power software [27]. Within the repeated measures design, to obtain power of 0.90 with alpha level set to 0.05 to observe an effect of *f* = 0.22 (20,21), the projected sample size was at least 58 people in total for the between-within group interactions.

Initially, we recruited approximately 50% more participants than indicated by the power analysis, as we anticipated a considerable number of drop outs and incomplete approach to training (expectation was based on former experience in recruitment of participants for studies involving ENT-related patients). Therefore, 42 patients and 44 healthy controls provided written informed consent and were invited to participate in the first session of the study. Patients were referred from general practitioners, ENT specialists, or neurologists. Of all the participants, those who did not complete both sessions and those who did not exceed 50% compliance ratio in “trigeminal training” were excluded from the study. This exclusion criterion was based on the hypothesis that to observe the effects of the “trigeminal training”, participants should undertake regular trainings with multiple trials every day, but at the same time we made a realistic assumption that participants might not comply with 100% of training. The final sample consisted of 35 patients (M_age_ = 58.4 years, SD = 14.8; Min_age_ = 21 years; Max_age_ = 79 years; 43% females; sinonasal disease (*n* = 23), idiopathic (*n* = 7), postinfectious (*n* = 3), congenital (*n* = 1) or posttraumatic (*n* = 1)) and 30 healthy controls (M_age_ = 36.7 years, SD = 14.5; Min_age_ = 20 years; Max_age_ = 73 years; 60% females). All studies were conducted at the Department of Otorhinolaryngology of the “Technische Universität Dresden”.

### Procedure

Participants were examined during two sessions. Within both sessions, the same measurements were taken. Both sessions were separated by “trigeminal training”. After the first session, each participant was equipped with three brown glass bottles (60 ml volume, height 65 mm, diameter of opening 35 mm) containing eugenol (clove-like smell, 10 ml of > 98% concentration), menthol (20 ml, 100 g menthol crystals in 100 ml propylene glycol) and acetic Acid (10 ml of 12.5%) soaked in a cotton pad to avoid spilling (all odors from Sigma-Aldrich, Deisenhofen, Germany). These substances were chosen on the basis of efficacy in trigeminal stimulation [28–30] and availability in the lab. Participants were asked to subsequently open the bottles and smell odors for 10 s, four times every day (in 3–4 h intervals) between the sessions. Mean duration of “trigeminal training” was 70 days. During the second session, participants were asked to estimate how many times a day (on average) they managed to perform training and how many days per week (on average). Based on this estimation, compliance ratio was calculated according to the formula: (declared average number of training per day/4 × declared average number of days with performed training per week/7). Additionally, all participants were asked to provide basic demographical information that could potentially influence olfactory functions (e.g., age, sex).

### Measurements

Objective measurements included trigeminal sensitivity to CO_2_ and lateralization abilities for eucalyptol. Participants’ trigeminal sensitivity was assessed using a CO_2_ stimulator [31]. A series of CO_2_ stimuli was presented to the participants through a standard bilateral nasal cannula. Stimulus duration increased by 50 ms from one stimulus to the other, starting at 100 ms until the subject felt a burning or stinging sensation in the nose and pushed a button to indicate that. After that stimulus, duration was decreased until the subject did not indicate a painful sensation by pressing the button anymore; and then the duration was increased again [31]. Maximum stimulus duration was 2000 ms. To ensure maximal reliability, this measurement was taken three times within each session, proceeded by a trial measurement. The final score represents the average value for the three measurements (in milliseconds) with lower scores indicating greater trigeminal sensitivity for CO_2_.

Lateralization abilities were quantified with the use of a mechanically operated stimulation device presenting a trigeminal stimulus—eucalyptol—to one of the nostrils, while the other was stimulated with odorless air (for details see: [4, 32]). After stimulation, participants were asked to indicate which nostril received the trigeminal stimulus, for a total number of 20 trials (maximum score of 20 points).

Subjective measures were based on self-rated nasal patency on ten-point Likert-type scales where “1” indicated very poor nasal patency and “10” indicated very good nasal patency.

### Statistics

All calculations were performed using IBM SPSS v. 24 software (SPSS Inc., Chicago, IL, USA) with the level of significance set to alpha = 0.05. To examine the influence of “trigeminal training” on the objective and subjective indicators of trigeminal function, we performed a series of Linear Mixed Models. Each model included group (patients vs healthy controls) and session (first vs second) as fixed factors; compliance and age were treated as the fixed covariates. Dependent variables included objective measurements, namely trigeminal sensitivity score and lateralization score, whereas subjective measurement was represented by the self-reported patency.

## Results

### Objective measurements

**Fig. 1:**
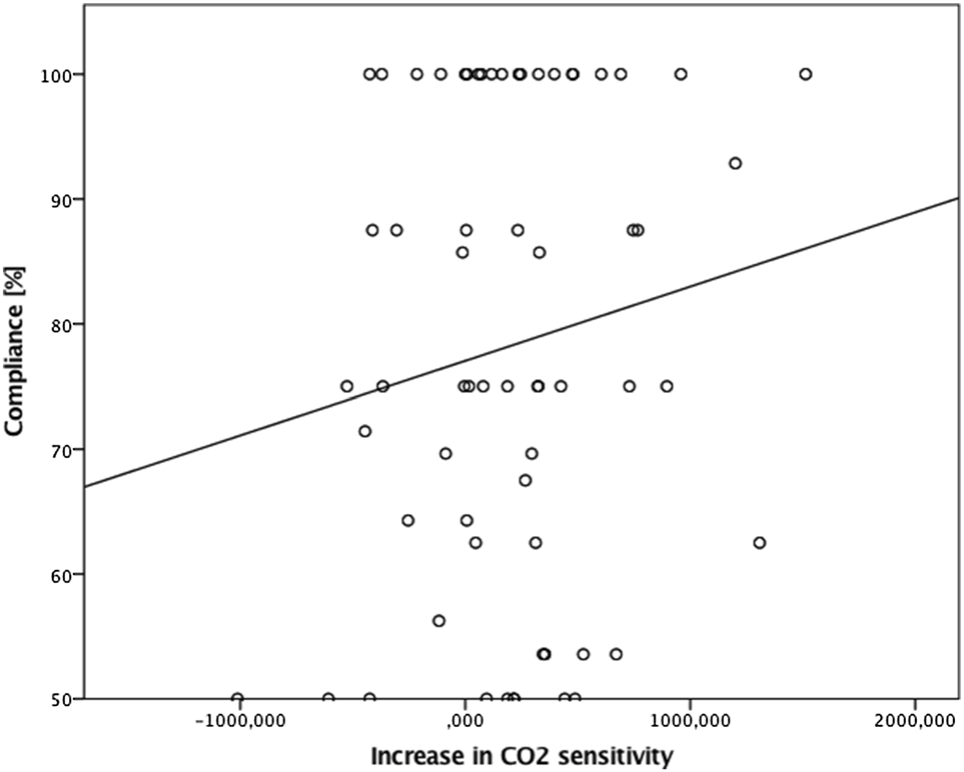
Relationship between compliance to the “trigeminal training” and increase in CO_2_ sensitivity calculated as a difference between CO_2_ sensitivity score obtained in the first session and CO_2_ sensitivity score obtained in the second session. Positive values represent decrease in stimuli duration that was perceivable to the participants during CO_2_ stimulation, whereas negative vales indicate that during the second session participants needed more time to experience trigeminal stimulation caused by CO_2_

Model performed for trigeminal sensitivity revealed a significant main effect of session for CO_2_ sensitivity, *F*(1,120) = 5, *p* = .027, showing that participants were significantly more sensitive during the second session [M = 780.4 ± 65.8 ms, 95% CI (649.8, 910.3)] in comparison to the first session [M = 990.9 ± 66.7 ms, 95% CI (857, 1122.2)] and a main effect of group, *F*(1,120) = 11.2, *p* = .001, confirming overall lower sensitivity to CO_2_ in patients [M = 1081.8 ± 73 ms, 95% CI (937.4, 1276.3)] than healthy controls [M = 688.3 ± 77.3 ms, 95% CI (535.2, 841.3)]. Compliance had a significant effect on the CO_2_ sensitivity, *F*(1,120) = 15.3, *p* < .001 (Fig. 1), while age was not a significant covariate.

**Fig. 2:**
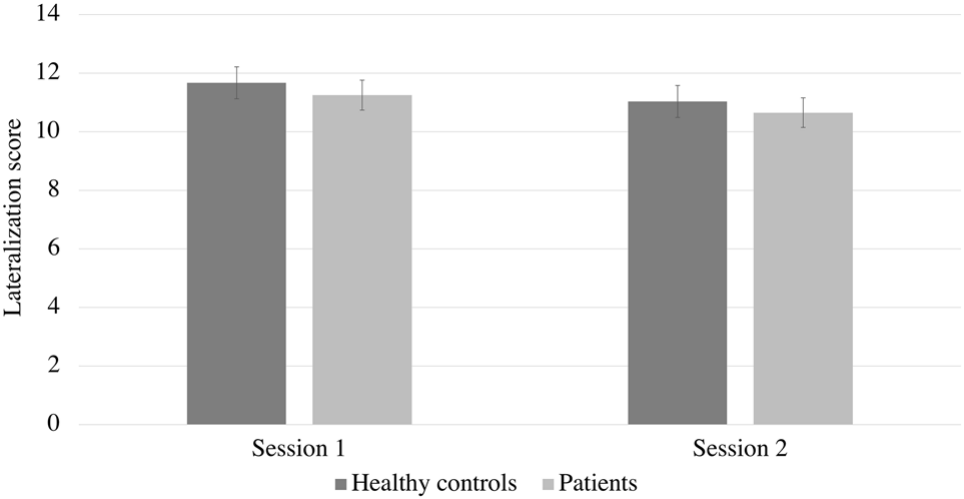
Mean scores for lateralization task across two sessions of “trigeminal training” in patients and healthy controls

No main or interaction effects of “trigeminal training” and group were found for lateralization scores (*F*s < 1.6, *p*s > .21; see Fig. 2).

### Self-rated nasal patency

**Fig. 3:**
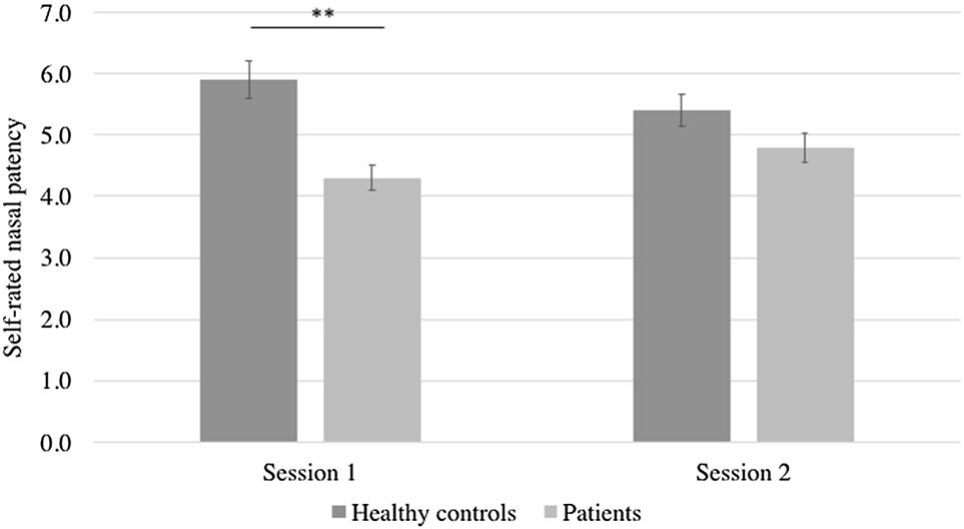
Self-rated nasal patency across two sessions (± standard error; **p < .01)

The model examining the influence of “trigeminal training” on self-rated nasal patency revealed a significant main effect of group, *F*(1,104) = 13.8, *p* < .001, indicating greater patency declared by healthy controls [M = 5.7 ± 0.21, 95% CI (5.3, 6.1)] compared to patients [M = 4.5 ± 0.18, 95% CI (4.2, 4.9)] and an interaction effect between the group and session factors *F*(1,104) = 4.7, *p* = .03, with pairwise comparisons suggesting that the initial difference between examined groups (*p* < .001) disappeared after “trigeminal training”, in the second session (*p* = .09). Participants’ age was a significant covariate of the self-reported nasal patency ratings, *F*(1,104) = 7.4, *p* = .008, indicating that the between-session difference in patency increased with the age of the participants (Fig. 3).

We analyzed the relationship between training duration and effects of “trigeminal training” by correlating the number of training days and the between-session difference in results (session II–session I). There was no significant relationship between training duration and trigeminal sensitivity (*p* = .31) and subjective nasal patency (*p* = .6), but there was a significant, negative relationship with lateralization (*r* = − .38, *p* = .002), but it disappeared after controlling for training compliance (*p* = .08). Effects of “trigeminal training” were not correlated with each other; however, there was a significant relationship between trigeminal sensitivity and subjective nasal patency in patients (*r* = .46, *p* = .014).

## Discussion

This study offers empirical evidence that exposure to intermitted trigeminal stimulation with various trigeminally active substances leads to an increase in trigeminal sensitivity in both patients and healthy controls, depending on their compliance to the “trigeminal training” regimen. Additionally, we observed an increase of the nasal patency in patients who underwent this procedure.

We found that “trigeminal training” had a positive influence on trigeminal sensitivity to CO_2_. This finding shows that the regular, short-lasting, intermittent exposure to trigeminal stimuli in low-concentration might be considered an effective “trigeminal training” aimed to increase trigeminal sensitivity. Our data offers empirical support for the claim that the effectiveness of such training may depend on compliance to the daily routine of trigeminal stimulation, underlining the importance of the regularity of training. The effects of “trigeminal training” on CO_2_ sensitivity in patients and healthy controls offer an optimistic perspective for clinical practice. It also suggests that despite the confirmed interaction between the olfactory and trigeminal systems [33–35], changes of trigeminal sensitivity remain independent from the functionality of the olfactory system.

“Trigeminal training” did not affect patients’ and healthy controls’ ability to lateralize eucalyptol. This is a rather unexpected finding, knowing that the trigeminal nerve, that has been regularly trained for approximately 2 months, is responsible for lateralization of odorants and registration of airflow direction [4, 15]. However, we did not observe any differences between patients and healthy controls as well as we did not observe any improvement after “trigeminal training”. Since means across groups and sessions varied between 10.7 and 11.7 points (wherein the expected level of chance would be 10 out of 20 trials), we speculate that answers provided by the participants were random. This might potentially result from fatigue and irritation of the trigeminal nerve caused by the preceding measurement of CO_2_ sensitivity. Therefore, for future studies investigating the effects of “trigeminal training”, we recommend to implement a limited number of trigeminal function indicators, or to separate the measurements by larger intervals.

We also found that the lateralization score was negatively correlated with the duration of trigeminal training, but the correlation became non-significant when controlling for training compliance. Overall, this pattern of results suggest that not the extent of the training, but rather the number of stimulations over a certain amount of time, may lead to trigeminal lassitude and habituation effects [25, 26].

Increased ratings of nasal patency in patients are promising indicators for the usefulness of “trigeminal training” in the treatment of patients complaining of nasal congestion. It corroborates former findings showing that trigeminal stimulation can bring a significant improvement to the subjective perception of the patients’ nasal airflow [7]. However, results of the current study confirm that the initial difference between the two examined groups observed in the first session disappeared in the second session, meaning that ratings of the patients became similar to those of controls. In this context, it is also important to note that the increase in patients’ subjective nasal patency between the two sessions tended to be significant (*p* < .1). However, the significant increase of subjective nasal airflow in patients might also reflect their expectations towards effectiveness of “trigeminal training”. Congested nose is a vexatious condition thus their expectations towards effectiveness of the procedure could have biased their self-reported nasal patency. On the other hand, the between-session difference in trigeminal sensitivity and subjective nasal patency was found to be significant only in the patients but not healthy controls, suggesting correspondence between psychophysical and self-reported data. These findings clearly await further investigation to understand the relationship between subjective and objective measurements of trigeminal function and their impact in the patients’ daily life. We also recommend further examination of the relationship between the cause of nasal obstruction and effectiveness of the “trigeminal training”. This should be the next step towards understanding of mechanisms underlying effective treatment.

One of the potential limitations of the current study refers to the age difference between controls and patients. Here, we observed that on average patients were over 20 years older than their healthy controls. It is commonly acknowledged that both olfactory [36] and trigeminal [37, 38] function decrease with age. Although we observed positive effects of “trigeminal training” in both groups, the efficiency of the “trigeminal training” in relation to age groups should be a matter of future interest with a special focus on replication of the current results with groups of more similar age. Having said that, according to the statistical analyses age had no influence on the current results. Equipping participants with diaries to note compliance on a daily basis could also be beneficial for the study procedure; however, we point to the fact that both diary and compliance declaration at the second session relate to the participants’ response bias. However, diary-driven data are less prone to memory distortion. Knowing that olfactory training is mostly effective in postinfectious patients [39], one could hypothesize a similar result in the case of “trigeminal training”. However, the current sample does not allow to determine the difference in effectiveness of “trigeminal training” with regard to the cause of olfactory loss due to the small and unequivocal numbers of patients of different etiologies.

To conclude, “trigeminal training” consisting of intermittent stimulation with diverse odorants leads to an increase of trigeminal sensitivity. Importantly, in patients, this training is also associated with an increase in self-rated nasal patency.
